# Dento–Osseous Variability of the Mental Foramen: A Retrospective CT-Based Morphometric Study

**DOI:** 10.3390/medicina62050871

**Published:** 2026-05-01

**Authors:** Andrei Urîtu, Alexandra Roi, Ciprian Roi, Doina Chioran, Alexandru Cătălin Motofelea, Ioana Riviș, Mircea-Alexandru Bălășoiu, Radu Dan, Mircea Riviș

**Affiliations:** 1 Doctoral School, University Clinic of Anesthesiology and Oral Surgery, Research Center of Dento-Alveolar Surgery, Anesthesia and Sedation in Dental Medicine, “Victor Babes” University of Medicine and Pharmacy, Eftimie Murgu Sq. No. 2, 300041 Timisoara, Romania; andrei.uritu@umft.ro (A.U.); mircea.balasoiu@umft.ro (M.-A.B.); 2 University Clinic of Oral Pathology, Multidisciplinary Center for Research, Evaluation, Diagnosis and Therapies in Oral Medicine, “Victor Babes” University of Medicine and Pharmacy, Eftimie Murgu Sq. No. 2, 300041 Timisoara, Romania; alexandra.moga@umft.ro; 3 University Clinic of Anesthesiology and Oral Surgery, Research Center of Dento-Alveolar Surgery, Anesthesia and Sedation in Dental Medicine, “Victor Babes” University of Medicine and Pharmacy, Eftimie Murgu Sq. No. 2, 300041 Timisoara, Romania; rivis.mircea@umft.ro; 4 Department of Internal Medicine, Faculty of Medicine, “Victor Babes” University of Medicine and Pharmacy, Eftimie Murgu Sq. No. 2, 300041 Timisoara, Romania; alexandru.motofelea@umft.ro; 5 Maxillofacial Surgeon, Rivis Dental Clinic, 307160 Dumbravita, Romania; badeaioanadaniela@yahoo.com; 6 Department of Surgery I, “Victor Babeș” University of Medicine and Pharmacy, 30041 Timisoara, Romania; radu.dan@umft.ro

**Keywords:** mandible, mental foramen, mental nerve, incisive nerve, tomography, X-ray computed, anesthesia, nerve block, retrospective studies, morphometric

## Abstract

*Background and Objectives:* This study aimed to identify osseous and dental mandibular landmarks that consistently indicate the location of the mental foramen, the primary reference for the mental–incisive trunk block. *Materials and Methods:* Computed Tomography (CT) scans of the mandibles of 100 patients from a Romanian population (N = 100) were retrospectively analyzed to measure the following: distances from the mental foramen to the basal border of the mandible, alveolar process/crest, and midline, as well as the position of the foramen relative to the lower premolars. These measurements were correlated with patients’ age (divided into three groups: 18–30 years, 30–60 years, >60 years) and gender. *Results:* The mental foramen was found to be closer to the alveolar crest (13.3 mm with SD = 3.3 in males and 10.7 mm with SD = 4.4 in females, overall mean = 12.3 mm, SD = 4.0) (*p* = 0.001) than to the inferior border of the mandible (14.3 mm with SD = 1.8 in males and 12.9 mm with SD = 1.6 in females, overall mean = 13.7 mm, SD = 1.9) (*p* < 0.001). In addition, the foramen was most frequently located adjacent to the second premolar (27.0%) rather than between the premolars (20.0%) and at a distance of ≃2.5 cm lateral to the midline (overall mean = 25.2 mm, SD = 3.5) (*p* = 0.034). *Conclusions:* Following the measurements performed, the mental foramen was identified as being closer to the alveolar crest in the vertical direction, at a distance of approximately 2.5 cm lateral to the midline, and most frequently located at the level of the second lower premolar.

## 1. Introduction

The inferior alveolar nerve is one of the three terminal branches of the posterior trunk of the mandibular nerve. Neurologically, it is classified as a mixed nerve, containing both sensory and motor fibers. The sensory fibers enter the mandibular canal via the mandibular foramen on the internal surface of the ramus, forming the proper inferior alveolar nerve. In contrast, the motor fibers contribute to the mylohyoid nerve, which innervates the mylohyoid muscle and the anterior belly of the digastric muscle [1].

After entering the ramus of the mandible, the sensory fibers descend vertically and anteriorly within the mandibular canal, alongside the inferior alveolar artery and vein, forming the inferior alveolar neurovascular bundle. At the level of the lower premolars, the mandibular canal bifurcates into a short mental canal, which exits at the mental foramen (mF), and an incisive canal that continues within the horizontal branch of the mandible toward the midline [2,3]. Accordingly, the neurovascular bundle divides into the mental branch, exiting through the mF, and the incisive branch [4], which provides sensory innervation to the bony chin and is associated with dento-periodontal structures [2,5].

Mental–incisive nerve (MIN) block is a representative peripheral trunk block technique for this region. While less commonly used due to its technical precision requirements, MIN block offers several advantages: simultaneous anesthesia of the mental and incisive nerves, fewer injections needed for extended procedures (such as serial extractions of lower anterior teeth or multiple endodontic treatments in one session), and the option of performing the block via endo-oral or exo-oral approaches depending on clinical needs.

Like other trunk block techniques, MIN block has limitations related to dentist/oral surgeon skills and detailed anatomical knowledge. Failure may occur due to variations in the position of the mF [2,4,6] relative to anatomical landmarks such as the alveolar process, inferior border of the mandible, mental symphysis and dento-periodontal units. Incorrect estimation of its position may lead to inadequate anesthesia, requiring repeated punctures, and may also increase the risk of iatrogenic injury to the neurovascular bundle. Correct needle placement and technique are critical, as the procedure is sensitive to puncture location and angle. In many cases, MIN block provides anesthesia only of the mental nerve, requiring additional expertise to achieve effective incisive nerve block when necessary.

However, despite its clinical relevance, the current literature provides heterogeneous and sometimes conflicting data regarding the precise anatomical position of the mental foramen across different populations, age groups, and sexes. This lack of consistency represents an important knowledge gap, particularly in relation to its impact on the predictability and failure rates of regional anesthesia in the mandible. This highlights the need for population-specific radiographic evaluations.

In this context, we hypothesize that the position of the mental foramen shows measurable variability influenced by age and sex, and that identifying consistent patterns relative to stable anatomical landmarks may improve the predictability of mental–incisive nerve block.

Therefore, the primary objective of our study was to determine the most frequent position of the mF in a Romanian population, while also assessing whether its relationship with anatomical landmarks (inferior border of the mandible, alveolar crest, mandibular midline and premolar–molar region) varies according to age and sex. The secondary objective was to identify clinically relevant positional patterns that may support more accurate and reliable mental–incisive nerve block.

## 2. Materials and Methods

The aim of this retrospective cross-sectional CT-based morphometric study was to evaluate the anatomical position of the mental foramen in a Romanian population and to assess its variability in relation to age, sex and adjacent anatomical landmarks. The study was approved by the Ethics Committee of the “Victor Babeș” University of Medicine and Pharmacy, Timișoara (approval reference number 09b from 24 February 2023). CT examinations were retrieved retrospectively from the institutional Picture Archiving and Communication System (PACS). The archive contains consecutive routine clinical examinations acquired for non-dental indications; no examinations were performed for the purposes of this study and no additional radiation exposure was incurred. All cranial/maxillofacial CT examinations archived between February 2023 and February 2024 were screened consecutively against the predefined inclusion and exclusion criteria, and every examination meeting these criteria was included; no random subsampling, frequency matching, or selection on demographic grounds was performed. The clinical indications for the included scans were head and maxillofacial trauma without mandibular involvement, suspected sinonasal or skull-base pathology, pre-operative ENT assessment, neurological investigation, vertigo work-up, and oncological staging or surveillance of head and neck regions other than the mandible. Examinations performed for orthodontic, implantological or other primarily dental indications were not present in this archive and were therefore not represented in the sample. Apart from sex and age, all patient identifiers were removed prior to analysis.

### 2.1. Inclusion and Exclusion Criteria

#### 2.1.1. A. Inclusion Criteria

- Patients of both sexes
- Patients who underwent a cranial CT scan for medical reasons related to the oro–maxillo–facial region, excluding those covered by the exclusion criteria below
- Patients aged 18–93 years (the upper age limit of 93 years reflected the maximum age of patients included in the available dataset during the study period and was not a predefined exclusion criterion)

#### 2.1.2. B. Exclusion Criteria

- Pregnant patients
- Patients under 18 years
- Patients with mandibular tumors or trauma-related pathologies (fractures of mandibular bone from any cause, whether traumatic or in pathological bone)
- Patients with contraindications to radiation
- Patients who had undergone surgical interventions for mandibular fracture reduction with osteosynthesis materials
- Patients whose CT images contained artifacts preventing accurate measurements between the analyzed mandibular landmarks.

No upper age restriction was applied, and no patients were excluded on the basis of age.

### 2.2. CT Measurements

This study was based on pre-existing CT scans acquired for medical non-dental indications. Therefore, conventional CT imaging was used as the imaging modality. CBCT was not available for the included patients.

#### 2.2.1. Measurement Protocol and Reproducibility

Scans were acquired using a Siemens Somatom Definition Edge (Germany) at 120 kV, with a slice thickness of 0.6 mm and an inter-slice distance of 0.4 mm. Three-dimensional reconstructions were performed using OpenRad Cloud (UK). Linear measurements conducted by two evaluators to minimize bias and potential errors were performed on 2D slices and 3D reconstructions. This combined approach enhances measurement accuracy and reproducibility. To address potential variability due to object orientation, all 3D models were standardized prior to measurement. The skulls were aligned according to anatomical reference planes, with the mandibular plane oriented parallel to the horizontal plane and the midsagittal plane aligned vertically. Measurements were performed using fixed, reproducible views, and evaluators were instructed not to alter orientation during measurement acquisition. When necessary, orthogonal slices were used to confirm point positioning. This protocol minimized errors related to viewing angle and ensured consistency across cases. Both evaluators underwent a calibration process prior to data collection. This included joint training sessions in which measurement protocols were reviewed and applied to a subset of cases not included in the final analysis. Measurements were repeated after a two-week interval to assess intra-examiner reliability. Inter-examiner reliability was also evaluated by comparing measurements between the two evaluators.

The Intraclass Correlation Coefficient (ICC) exceeded 0.75. This indicates good to excellent agreement and confirms the reliability of the measurement protocol.

#### 2.2.2. Anatomical Reference Points

- The mental foramen (mF)—defined as the opening located on the anterolateral surface of the mandible
- The inferior border of the mandible—defined as the most inferior margin of the mandibular body
- The superior border of the alveolar process/crest—defined as the most coronal aspect of the alveolar bone
- The mandibular midline—defined as the vertical line passing through the mandibular symphysis

### 2.3. Primary and Secondary Outcomes

The primary outcome of the study included measurements between the anatomical reference points (expressed in millimeters and illustrated in the figures below). The analyzed distances:

- The distance mF—inferior border of the mandible
- The distance mF—superior border of the alveolar process/crest
- The distance mF—midline (anatomically defined by the anterior point of the mental symphysis)

In addition, the position of the mF was evaluated in relation to the teeth present on the dental arch (between the two lower premolars, at the level of the first or second lower premolar, mesial to the first premolar, distal to the second premolar, or without a dento-periodontal counterpart in fully or partially edentulous patients). In cases where no dento-periodontal reference was present, the position of the mental foramen was recorded as “0” (no dental counterpart) and analyzed as a separate category. These patients were not excluded from the study, since the mental foramen is a stable anatomical structure independent of dental presence. This classification was based on previously described anatomical landmarks in the literature, with minor adaptations to fit the radiographic evaluation used in this study.

The secondary outcome of the study included the analysis of differences in these measurements according to age and sex.

**Figure 1:**
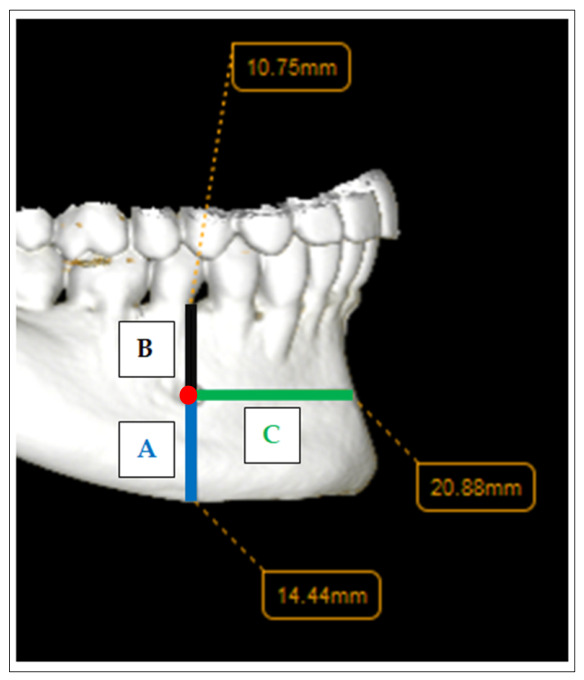
Measurements on the external surface of the horizontal branch of the mandible (**A**: mF—inferior border of the mandible distance; **B**: mF—superior border of the alveolar process/crest distance; **C**: mF—midline distance).

**Figure 2:**
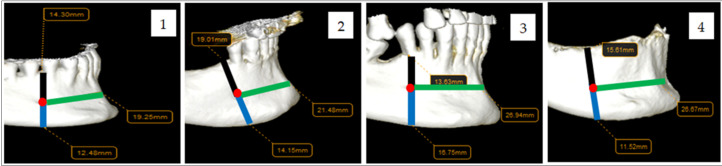
The position of the mF in relation to the dental units present on the arch ((**1**)—between the two lower premolars; (**2**)—at the level of the first premolar; (**3**)—at the level of the second premolar; (**4**)—without a dento-periodontal counterpart).

All these details are illustrated in the figures below (Figure 1 and Figure 2).

### 2.4. Statistical Analysis

Data were entered into Microsoft Excel and analyzed using RStudio (2025.09.0). The statistical analysis aimed to assess differences in mandibular measurements and their correlation with patient sex and age. Descriptive analysis (Mean, Standard Deviation (SD), Range, Median and Interquartile Range [IQR]) by gender was computed for each measurement from the mF to the inferior border of the mandible, the superior border of the alveolar process/crest, and midline. Furthermore, Mean, SD and Range were calculated for the position of the mF in relation to the dental units. Descriptive analysis by age groups was performed using the same approach. Each variable was tested for normality using the Shapiro–Wilk test. Power analysis confirmed that the study had sufficient statistical power to detect measurement differences. The sample of 100 participants was considered sufficient to detect medium-sized effects. An unpaired *t*-test was used to compare measurements between genders, while the Mann–Whitney U test was used for not normally distributed variables.

The sample was divided into three age groups: 18–30 years (G1), 30–60 years (G2), and over 60 years (G3). The Analysis of Variance (ANOVA) test was used to assess differences among age groups. For variables not meeting the homogeneity of variance assumption, Welch’s ANOVA test was applied. Post hoc comparisons to identify specific group differences were performed using Tukey’s Honestly Significant Difference (HSD) test. This test was applied when ANOVA indicated significant results. Measurement consistency was confirmed using the Intraclass Correlation Coefficient, which exceeded 0.75. A significance level of 0.05 was applied for all tests, and *p*-values below 0.05 were considered statistically significant.

This study followed the general principles of the STROBE guidelines.

## 3. Results

Among the total participants, 39 were females (N = 39) and 61 were males (N = 61). The mean age was 63.1 years (SD = 21.5) for females and 47.4 years (SD = 17.9) for males. Overall mean = 53.5 years (SD = 20.7) (*p* < 0.001). Age ranged 22–93 years in females and 18–83 years in males. Below, Table 1 shows the mean and standard deviation of mF position on the external surface of the mandibular body.

According to the results, regarding the vertical position of the mF, it is located closer to the superior border of the alveolar process than to the basal border of the mandible. In relation to its distance from the midline, the mF is positioned approximately 2.5 cm laterally on the anterolateral surface of the mandible.

**Figure 3:**
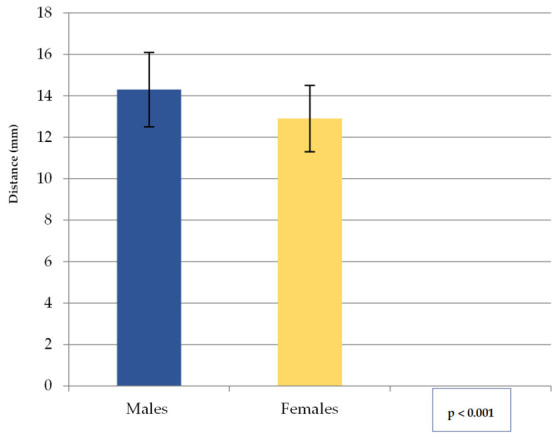
The mean mF—inferior border of the mandible distance (in mm) by gender. Note: Columns represent Mean, Error bars represent SD.

The values obtained for the distance mF—inferior border of the mandible (A) (mean, SD) are shown in Figure 3. The overall mean was 13.7 mm (SD = 1.9). The range was 10.2–17.7 mm for males and 9.8–15.9 mm for females, with an overall range of 9.8–17.7 mm. These results indicate that in males, the mF is positioned more cranially relative to the inferior border of the mandible than in females.

Median and IQR for mF—inferior border of the mandible distance are indicated in Table 2.

**Figure 4:**
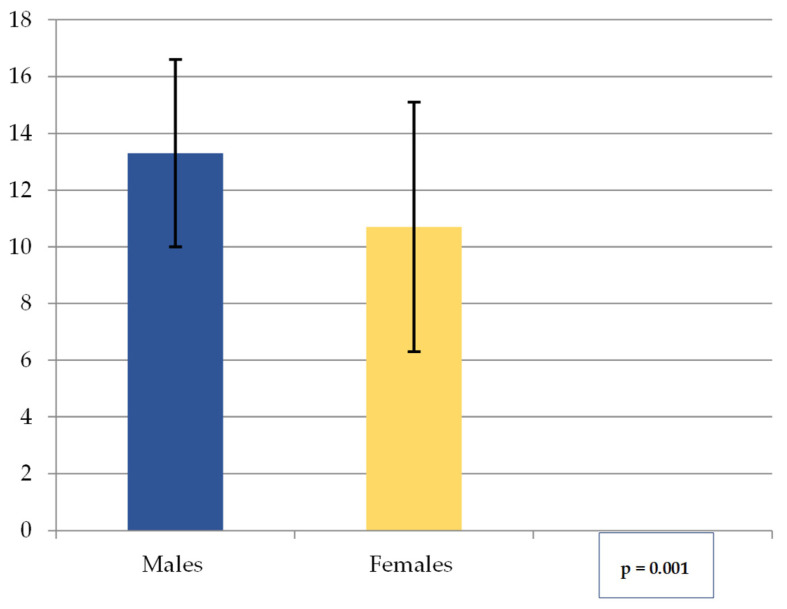
The mean mF—superior border of the alveolar process/crest distance (in mm) by gender. Note: Columns represent Mean, Error bars represent SD.

The values obtained for the distance mF—superior border of the alveolar process/crest (B) (mean, SD) are shown in Figure 4. The overall mean was 12.3 (SD = 4.0). The range was 2.8 to 19.9 mm for males and 0.0 to 16.5 mm for females, with an overall range of 0.0 to 19.9 mm. These results show that in males, the mF is located more caudally relative to the superior border of the alveolar process than in females.

Median and IQR for mF—superior border of the alveolar process/crest distance are indicated in Table 3.

Considering both measurements, the position of the mF relative to the two landmarks (the inferior border of the mandible and the superior border of the alveolar process) can be described as central—slightly lower in males and higher (closer to the alveolar process) in females.

**Figure 5:**
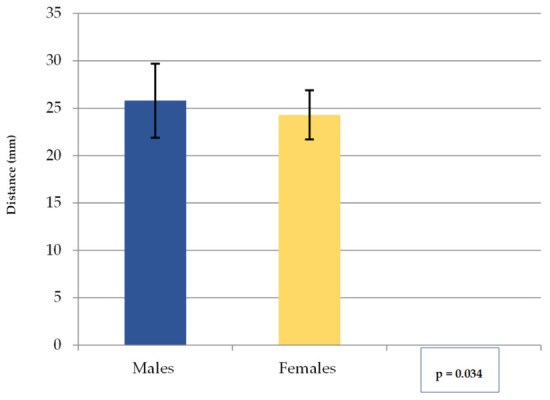
The mean mF—midline distance (in mm) by gender. Note: Columns represent Mean, Error bars represent SD.

The values obtained for the distance mF—midline (C) (mean, SD) are shown in Figure 5. The overall mean was 25.2 (SD = 3.5). The range was 18.7 to 45.5 mm for males and 19.2 to 31.3 mm for females, with an overall range of 18.7 to 45.5 mm. In the current analysis, the mF is located approximately 2.5 cm laterally from the midline on the anterolateral surface of the mandibular body, with the note that in males it is positioned slightly more lateral.

Median and IQR for mF—midline (C) distance are indicated in Table 4.

Regarding the position of the mF in relation to dental units, its location was evaluated using the following dento-periodontal units as reference: the lower canine, mandibular premolars, and the first lower molar. The position of the mF was analyzed by sex and organised in the following categories: mesial to the first lower premolar (*m-pm1*), at the level of the first lower premolar (*pm1*), at the level of the second lower premolar (*pm2*), between the two lower premolars (*pm1-pm2*), and distal to the second lower premolar (*d-pm2*). In cases where the position of the mF could not be related to any dento-periodontal unit (due to tooth loss), it was recorded as *0*.

**Figure 6:**
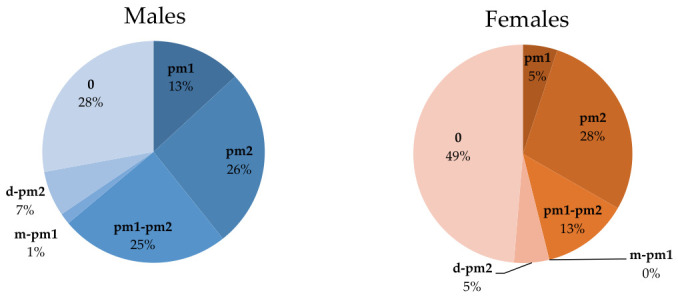
Percentage distribution of mF positions in relation to dental units by gender.

The positions of the mF in relation to the dental units by gender are presented in Table 5 and Figure 6. The most frequent positions were at *pm2* (27% of cases) and at *pm1-pm2* (20% of cases).

Overall, with a *p*-value of ≃0.40, the comparative analysis by sex did not reveal statistically significant differences in the position of the mF relative to the teeth.

The participants had a mean age of 47.4 years (SD = 17.9) for males and 63.1 years (SD = 21.5) for females, resulting in an overall mean of 53.5 years (SD = 20.7) (*p* < 0.001). Male participants ranged in age from 18 to 83 years, whereas female participants ranged from 22 to 93 years.

The position of the mF in relation to anatomical landmarks (the inferior border of the mandible, the superior border of the alveolar process/alveolar crest, and the midline) is also influenced by the age of the patients, divided into three reference groups: G1, G2, and G3.

Considering the data summarized in Table 6, the distance from the mF to the inferior border of the mandible (A) decreases after the age of 60. The overall mean for the distance mF—superior margin of the alveolar process/crest (B) was 12.3 mm. The difference is highly statistically significant (*p* < 0.001) and demonstrates a clear decrease in this distance with advancing age, particularly after 60 years. The mechanism involved is likely physiological in nature and may be associated with osteolytic changes secondary to the loss of dento-periodontal units. Moreover, while the two distances described above change with age, the position of the mF relative to the midline (C) remains relatively constant.

The position of the mF within the three age groups was also analyzed in relation to the previously established dental landmarks. Overall, the *p*-value was <0.001. This indicates that the comparative analysis by age groups reveals statistically significant differences in the position of the mF relative to the dental units.

**Figure 7:**
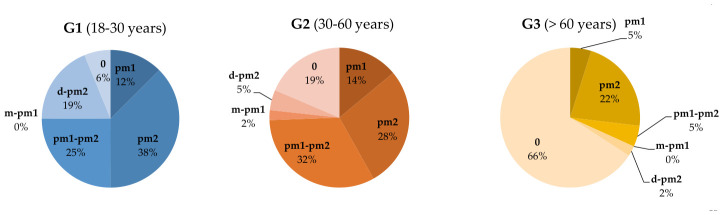
Percentage distribution of mF positions in relation to dental units by age groups.

In G1, the mF is mostly located at the level of *pm2* or *pm1-pm2*. In G2, the situation appears to be similar, while cases without a dental reference point increase. In G3, most cases no longer have a dental reference point, making position 0 the dominant category. Additionally, extreme positions like *m-pm1* and *d-pm2* are rare in all age groups (especially in G3). Similar to the position of the mF in relation to dental units by sex, the most frequent positions of the mF across the age groups were at *pm2* (27% of cases) and *pm1-pm2* (20% of cases). The data are summarized in Table 7 and Figure 7.

Considering all the measurements above, the position of the mF can be described as central—slightly lower in males and higher (closer to the alveolar process) in females. Furthermore, the mF is located approximately 2.5 cm laterally from the midline on the anterolateral surface of the mandibular body, slightly more lateral in males. The comparative analysis by sex did not reveal statistically significant differences in the position of the mF relative to the teeth. The distance from the mF to the superior border of the mandible decreases after 60 years. On the other hand, the position of the mF relative to the midline remains relatively constant.

## 4. Discussion

Despite multiple previous imaging studies, there is still a lack of population-specific data regarding the combined vertical variability and transverse stability of the mF, particularly in relation to its clinical implications for regional anesthesia techniques. Our study shows that, in relation to anatomical landmarks, the mF is located closer to the superior border of the alveolar process than to the inferior border of the mandible and is positioned at a lateral distance of ≃2.5 cm from the midline.

A comparable and relevant finding can be identified in the study by Abdullah Ebrahim Laher and Zeyn Mahomed, published in 2016. Using ultrasonographic assessment of the position of the mF in a cohort identical in size to ours (100 patients), the authors concluded that, when referenced to the superior border of the alveolar process and the inferior border of the mandible, the mF is situated more cranially [7].

The closer proximity of the mF to the superior border of the alveolar process is further supported by the interpretation of measurements reported by Ebad Mallahi et al. (2024) [2] in their study. In this study, mean values of ≃11.2 mm were recorded for the distance between the mF and the alveolar process, while a distance of ≃13.5 mm was noted between the mF and the inferior border of the mandible.

Sex-related differences in the distances between the mF and various anatomical landmarks have been reported in several studies. In a cohort of 100 patients, Filiz Direk et al. (2018) [8] analyzed Multidetector Computed Tomography (MDCT) images to determine the position and number of lingual vascular canals in the mandible, as well as the position of the mF. The authors concluded that the distances between the mF and the established reference points (except for the posterior landmark) were greater in males than in females [8]. This observation is partially supported by our own measurements, which indicate that in males the mF is positioned slightly closer to the basal border of the mandible, as reflected by a greater distance from the superior reference point.

Using a substantially larger sample comprising 162 females and 139 males, Dilek Coban et al. (2025) evaluated Cone Beam Computed Tomography (CBCT) images to assess both the mF and accessory mental foramen. With the superior, inferior, and posterior mandibular borders, as well as the midline, serving as reference landmarks, the authors concluded that in males, distances such as those between the mF and the posterior border and between the mF and the inferior border were bilaterally greater than those observed in females [9].

The study conducted by Beenish Fatima Alam et al. (2024) [10] follows a similar line of research. The reported results indicate higher values in males for measurements performed between the mF and the inferior border, the alveolar crest, and the anterior mandible within the studied population. Furthermore, in their study, Michaela Cellina et al. (2023) revealed that the distances from the superior and inferior borders to the mF were higher in males than in females [11].

According to age groups, our measurements indicate that the position of the mF changes with increasing age. A reduction in the distance from the inferior border is observed, which is counterintuitive compared to the general trend and data reported in the literature [12], while a decrease in distance from the superior border of the alveolar process follows the expected pattern. The position of the mF relative to the midline remains relatively constant (*p* = 0.464).

This passive cranial shift may result from the loss of dento-periodontal units and subsequent alveolar bone remodeling. However, this cannot be directly confirmed in the present study and should therefore be interpreted as a plausible explanatory mechanism rather than a demonstrated causal relationship. The differences observed in our study were particularly significant in the G3 group (*p* < 0.001). In many cases, the mF may even be located on the crest of the edentulous alveolar ridge, which can considerably complicate both prosthetic and surgical treatments.

A key finding of our study is the relative stability of the distance between the mF and the mandibular midline, regardless of age. Unlike vertical measurements, which may be affected by alveolar bone remodeling, the transverse position remains largely unchanged. This suggests that the midline may represent a more reliable anatomical landmark for clinical orientation, particularly in cases where vertical reference points are altered, such as in elderly or edentulous patients.

The distance of ≃2.5 cm from the midline is further supported by the study published by Alma Voljevica et al. (2015) [13]. The authors analyzed the position of the mF in 150 dry human mandibles by calculating vertical and transverse distances relative to specific anatomical landmarks. The distance between the anterior border of the mF and the mental symphysis demonstrated an average value of ≃25.6 mm on both the left and right sides [13]. This observation aligns with the findings of Mahnaz Sheikhi and Mitra Karbasi Kheir (2016) [12]. The authors reported mean distances of ≃25 mm (25.8 mm on the right side and 25.6 mm on the left) between the mF and the midline, based on 180 CBCT measurements.

According to most anatomical references, the mF is typically located on the anterolateral surface of the mandibular body [3], midway between the inferior border of the mandible and the superior border of the alveolar process, superior to the initial portion of the external oblique line, and along the vertical line connecting the three cranial foramina (supraorbital, infraorbital, and mental). In relation to soft tissues, the mF is positioned ≃0.5 cm medial to the midpupillary line. With respect to the mandibular premolar group, the most frequent position of the mF is between the two mandibular premolars or directly in line with the second premolar [3].

Based on these data, as well as the findings from our study and the previously cited literature in this article, there is significant variability in the position of the mF relative to the classical landmarks in many cases. This variability may increase the risk of failure when performing a MIN peripheral trunk block due to incorrect selection of the puncture site. We consider it highly advisable to adapt the classical anesthesia technique to every individual patient. The clinician’s adjustment should be grounded in a thorough understanding of the anatomical landmarks and, depending on the individual clinical situation, may incorporate insights from the specialized literature, namely: the mF being located ≃2.5 cm lateral to the midline [13] and cranio-caudal variability relative to the alveolar process and the inferior border.

Our study shows that the most frequent position of the mF, both by sex and by age group (G1, G2, G3), is at the level of the *pm2* (27%), followed by *pm1-pm2* (20%). The third most frequent position is *pm1* (10%). Comparative analysis by sex did not reveal statistically significant differences (*p* ≃ 0.40), whereas the comparative analysis across age groups showed a significant difference (*p* < 0.001).

Michaela Cellina et al. (2023) analyzed 100 CBCT images with an equal distribution of sexes. Regarding the position of the mF relative to dental landmarks, the authors emphasized that the most frequent position of the mF is between the two mandibular premolars, followed by the position aligned with the axis of the second premolar [11]. Similar findings were observed by Algabri et al. (2025) [14], even though they assessed the mF position using panoramic radiographs. Analyzing the mF position in both vertical and horizontal dimensions, they concluded that the most frequent horizontal position is between the two mandibular premolars, followed by the position corresponding to the axis of the second premolar. In our study, these two positions are reversed. Nevertheless, in both our study and the aforementioned study, no statistically significant differences were reported between sexes.

Thus, in addition to the anatomical landmarks used for MIN peripheral trunk block, the position of the mF in relation to the region of the mandibular premolars may also be considered. Beyond its vertical cranio-caudal variability and its distance of ≃2.5 cm from the midline, particular attention may be paid to the location of the mF, which is most often found either between the two mandibular premolars or at the level of the second mandibular premolar. Knowledge of these frequent locations may play a significant role in choosing the optimal puncture site to achieve greater accuracy in peripheral trunk anesthesia or in modifying the position of the oral mucosal incisions during surgery. On the other hand, the relatively high proportion of cases without a dental reference point in our study highlights the limitations of relying solely on dento-periodontal landmarks. This is particularly relevant in older populations, where tooth loss is more prevalent. Consequently, clinicians should integrate both dental and osseous landmarks when estimating the position of the mental foramen, rather than depending exclusively on tooth-related references.

Given the anatomical variability, radiographic or imaging assessment becomes increasingly important for the success of dental or surgical procedures requiring anesthesia of the incisive and mental nerves. Radiographic or CBCT assessment is primarily used for precise localization of the mF, which is often not visible on panoramic radiographs or can be mistaken for a radiolucency corresponding to chronic apical periodontitis of the mandibular premolars. In such cases, CBCT can provide valuable additional information. Furthermore, accessory mental foramina, sometimes identified on CBCT [9], may complicate the achievement of effective anesthesia. Measurement of distances on CBCT between the mF and the vertical landmarks described in this study may help guide the selection of the optimal depth for the anesthetic puncture. Moreover, an mF located more anteriorly can inform clinicians whether the puncture should be performed anterior to the classic reference point (the vestibular gingival mucosa of the first molar) or at this point but using a longer needle to ensure that the tip reaches the foramen. To increase the accuracy rate of the MIN block technique, we recommend the intraoral anesthetic puncture be performed at the level of the first mandibular molar, with an anterior, medial, and inferior needle trajectory.

For isolated Mental nerve (MN) block, administration of a minimal amount of anesthetic solution near the mF may be sufficient. When anesthesia of the incisive nerve is also required using the MIN block technique, it is recommended that the needle tip be advanced minimally into the mental canal.

Finding some atypical positions of the mF could help clinicians adjust their approach, ensuring the anesthetic effectively reaches the target nerves. This reduces the risk of anesthesia failure, allowing many dental and surgical procedures to be successfully performed within the area innervated by these nerves: dental and endodontic treatments of the lower anterior teeth, extraction of the lower anterior teeth, excisions of tumors in the gingival mucosa, labial mucosa or labio-mental skin, and minor salivary gland biopsies. Furthermore, intraoperative incidents and post-anesthetic complications could be avoided, minimizing patient discomfort and enhancing procedural predictability. One of the most commonly reported adverse events is direct injury to the mental nerve sheath or needle-induced trauma to the neurovascular bundle. Postoperative complications may include paresthesia [15] or residual anesthesia within the mental nerve distribution (lower lip, labial mucosa, vestibular gingiva from mF to midline and lower teeth from mF to central incisor) [16].

Our study has several limitations. First, its retrospective cross-sectional CT-based morphometric design limits control over potential confounding variables and restricts causal inferences. In addition, the lack of multivariate analysis does not allow assessment of the independent effects of age and sex on the position of the mF. The observed age imbalance between male and female groups may also act as a potential confounding factor in sex-based comparisons. Second, selection bias may be present due to the use of a single-center dataset and the characteristics of the available CT records. Third, measurement bias cannot be completely excluded, as variations in CT scan resolution may have influenced the accuracy of linear measurements.

Overall, the findings should be interpreted as population-specific anatomical reference data rather than universally applicable clinical thresholds.

## 5. Conclusions

Anatomical and radiographic data provide an important framework for understanding and performing a peripheral trunk block.

The mF, often located midway between the superior border of the alveolar process and the inferior border of the mandible, should be approached with flexibility by clinicians. Its position relative to the midline should also be considered (≃2.5 cm laterally from the midline), especially since the distance between these points tends to remain stable despite vertical changes that occur with age. Knowledge of medical information, accurate interpretation of paraclinical examinations, and a precise objective clinical assessment may guide optimal administration of anesthesia.

Given the relatively limited number of studies addressing this topic, our findings are consistent with the existing evidence to date but emphasize the critical importance of a thorough understanding of each individual clinical case. Within the limitations of this study, the results should be interpreted as population-specific reference data.

## Figures and Tables

**Table 1:** Descriptive analysis of the position of the mF on the horizontal branch of the mandible.

Distance (in mm) Between the mF and Anatomical Landmarks	Mean (SD)	Median [IQR]
mF—Inferior border (A)	13.7 (1.82)	13.8 [12.4–15.1]
mF—Alveolar Process/Crest (B)	12.3 (3.81)	13.1 [10.2–15.4]
mF—Midline (C)	25.2 (3.52)	25.1 [23.1–27.2]

Abbreviations: mF—Mental foramen; SD—Standard Deviation; IQR—Interquartile Range.

**Table 2:** Median and IQR values for mF—inferior border of the mandible (A) distance.

GROUP	Median (mm)	[IQR] (mm)
Males (N = 61)	14.2 mm	[13.1–15.5] mm
Females (N = 39)	12.9 mm	[11.8–14.0] mm
Overall (N = 100)	13.8 mm	[12.4–15.1] mm

Abbreviations: IQR—Interquartile Range.

**Table 3:** Median and IQR values for mF—superior border of the alveolar process/crest (B) distance.

GROUP	Median (mm)	[IQR] (mm)
Males (N = 61)	13.5 mm	[11.1–15.5] mm
Females (N = 39)	11.5 mm	[7.8–14.2] mm
Overall (N = 100)	13.1 mm	[10.2–15.4] mm

Abbreviations: IQR—Interquartile Range.

**Table 4:** Median and IQR values for mF—midline (C) distance.

GROUP	Median (mm)	[IQR] (mm)
Males (N = 61)	25.6 mm	[23.2–28.1] mm
Females (N = 39)	24.2 mm	[22.5–26.0] mm
Overall (N = 100)	25.1 mm	[23.1–27.2] mm

Abbreviations: IQR—Interquartile Range.

**Table 5:** The position of the mF in relation to the dental units by gender.

The Position of mF	Males (N = 61)	Females (N = 39)	Total (N = 100)	Total (%)
pm1	8	2	10	10%
pm2	16	11	27	27%
pm1-pm2	15	5	20	20%
m-pm1	1	0	1	1%
d-pm2	4	2	6	6%
0	17	19	36	36%

Abbreviations: mF—Mental foramen; N—Number of participants; pm1—First lower premolar; pm2—Second lower premolar; pm1-pm2—Between the lower premolars; m-pm1—Mesial to the first lower premolar; d-pm2—Distal to the second lower premolar; 0—Position of mF not related to any dento-periodontal unit.

**Table 6:** Association between age and position of the mF in relation to anatomical landmarks.

Indicator	Groups/N			Overall/Total N	*p* Value
	G1/N = 16	G2/N = 43	G3/N = 41	N = 100	
mF—Inferior Border (mm)	**A**				*p* = 0.002
Mean (SD)	13.9 (1.9)	14.4 (1.7)	13.0 (1.8)	13.7 (1.9)	
Range Median IQR	11.0–17.6 13.8 12.7–15.1	10.8–17.4 14.3 13.2–15.6	9.8–17.7 13.1 11.9–14.2	9.8–17.7 13.8 12.4–15.1	
mF—Alveolar process/Crest (mm)	**B**				*p* < 0.001
N—Miss	0.0	0.0	1.0	1.0	
Mean (SD)	14.9 (2.0)	13.3 (2.7)	10.1 (4.6)	12.3 (4.0)	
Range Median IQR	12.3–18.1 14.8 13.5–16.3	7.4–19.9 13.5 11.4–15.2	0.0–19.0 11.2 6.8–13.5	0.0–19.0 13.1 10.2–15.4	
mF—Midline (mm)	**C**				*p* = 0.464
N—Miss	0.0	0.0	2.0	2.0	
Mean (SD)	25.1 (3.0)	25.7 (4.3)	24.8 (2.7)	25.2 (3.5)	
Range Median IQR	19.2–30.2 25.0 23.4–26.8	18.7–45.5 25.5 23.1–28.3	19.7–31.3 24.7 23.0–26.6	18.7–45.5 25.1 23.1–27.2	

Note: One-way ANOVA test was used for comparisons between age groups. Abbreviations: mF—Mental foramen; N—Number of participants; SD—Standard Deviation; G1—Group 1; G2—Group 2; G3—Group 3; *p*—Statistical significance, IQR—Interquartile Range.

**Table 7:** The position of the mF in relation to the teeth by age groups.

The Position of mF	G1 (N)	G2 (N)	G3 (N)	Total (N)
pm1	2	6	2	10
pm2	6	12	9	27
pm1-pm2	4	14	2	20
m-pm1	0	1	0	1
d-pm2	3	2	1	6
0	1	8	27	36

Abbreviations: mF—Mental foramen; N—Number of participants; pm1—First lower premolar; pm2—Second lower premolar; pm1-pm2—Between the lower premolars; m-pm1—Mesial to the first lower premolar; d-pm2—Distal to the second lower premolar; 0—Position of mF not related to any dento-periodontal unit; G1—Group 1; G2—Group 2; G3—Group 3.

## Data Availability

The data presented in this study are available on request from the corresponding author. Data are not publicly available due to privacy restrictions.

## Abbreviations

The following abbreviations are used in this manuscript:

CT	Computed Tomography
N	Number of participants
mF	Mental foramen
MIN	Mental Incisive nerve
2D	Bidimensional
3D	Tridimensional
SD	Standard Deviation
G1	Group 1
G2	Group 2
G3	Group 3
ANOVA	Analysis of Variance
*p*	Statistical significance
IQR	Interquartile Range
mm	Millimeters
m-pm1	Mesial to the first lower premolar
pm1	First lower premolar
pm2	Second lower premolar
pm1-pm2	Between the lower premolars
d-pm2	Distal to the second lower premolar
0	Position of mF not related to any dento-periodontal unit
MDCT	Multidetector Computed Tomography
CBCT	Cone Beam Computed Tomography
MN	Mental nerve
